# The generalized Vogel-Fulcher-Tamman equation for describing the dynamics of relaxor ferroelectrics

**DOI:** 10.1038/s41598-019-48864-0

**Published:** 2019-08-27

**Authors:** Rafael Levit, Julio C. Martinez-Garcia, Diego A. Ochoa, José E. García

**Affiliations:** 1 Department of Physics, Universitat Politècnica de Catalunya - BarcelonaTech, 08034 Barcelona, Spain; 2 Group of Nanomaterials and Microsystems, Department of Physics, Universitat Autònoma de Barcelona, 08193 Bellaterra, Spain

**Keywords:** Ferroelectrics and multiferroics, Materials for devices

## Abstract

Relaxor ferroelectrics (RF) are outstanding materials owing to their extraordinary dielectric, electromechanical, and electro-optical properties. Although their massive applications, they remain to be one of the most puzzling solid-state materials because understanding their structural local order and relaxation dynamics is being a long-term challenge in materials science. The so-called Vogel-Fulcher-Tamman (VFT) relation has been extensively used to parameterize the relaxation dynamics in RF, although no microscopic description has been firmly established for such empirical relation. Here, we show that VFT equation is not always a proper approach for describing the dielectric relaxation in RF. Based on the Adam-Gibbs model and the Grüneisen temperature index, a more general equation to disentangle the relaxation kinetic is proposed. This approach allows to a new formulation for the configurational entropy leading to a local structural heterogeneity related order parameter for RF. A new pathway to disentangle relaxation phenomena in other relaxor ferroics could have opened.

## Introduction

Materials are usually ordered at low temperatures (e.g., crystals) whereas they show disordered states at high temperatures (e.g., liquids). The order degree is controlled by the presence or absence of correlations between the material entities (e.g., molecules, electrical or magnetic dipoles, colloidal particles, etc.) upon the change of a control physical parameter (e.g., temperature, pressure, or density). However, there are systems where the disordered state remains at low temperatures such as glass materials (GM), which are non-equilibrium amorphous (disordered) systems with no long-range translational order (periodicity).

GM has a tremendous impact for technological applications showing optimal properties in comparison with their crystalline counterparts^1,2^. For instance, (i) many electrical transmission systems are made with glass optical fibers transmitting optical signals longer than crystalline materials, which is fundamental for improving the efficiency of the telecommunication network, (ii) electrical transformers fabricated with metallic glasses minimize more effectively the electrical losses in comparison to polycrystalline metals, thereby becoming metallic glasses optimal materials for improving the power generation and power transmission of electricity, (iii) in the pharmaceutical industry, glasses shown a better bioavailability than their crystalline counterpart, which suppose higher solubility and therefore lower doses of the active principle are required. Furthermore, since GM are out of equilibrium materials, their process of formation can be tunneled to produce composite materials (e.g., glass-filled polymers) with significant physical properties (electrical conductivity, mechanical sthength, rigidity, etc.) with have strong industrial applications^2^. When the physical control parameter is the temperature, GM can be obtained by quenching a liquid sufficiently fast enough to avoid crystallization passing through an intermediate state, defined as super-cooled liquid (SCL), to a glass low-temperature state^3^.

Relaxor ferroelectrics (RF) are another example of systems maintaining the disordered (quenched) state at ‘low temperatures’. RF are functional materials considered to bear a revolutionary potential for a myriad of modern electronic applications due to their unique properties such as ultrahigh strain and outstanding piezoelectric behavior (hysteresis-free electromechanical response)^4^, excellent electroacoustic response^5^, and remarkable electro-optic properties^6^. RF, mostly perovskite-structured (general formula, ABO_3_), are compositionally disordered systems where the arrangement of different ions on equivalent crystallographic sites (A or B) is partially or fully disordered^7^. A typical example of canonical RF is the Pb(Mg_1/3_Nb_2/3_)O_3_ (PMN) systems where the non-isovalent ions Mg^2+^ and Nb^5+^ are fully or partially disordered on the B-site^6^. The PMN solid solution with small concentration of PbTiO_3_ (PMN-PT) also shows relaxor behavior but, in this case, besides the non-isovalent ions disorder, the inhomogeneous chemical distribution of Ti^4+^ also contributes to the disorder of the system. Relaxor behavior can also be found in homovalent solid solutions like Ba(Zr_x_Ti_1−x_)O_3_ (BZT) or in non-stoichiometric solid solutions such as the Pb_1−x_La_x_(Zr_1−y_,Ti_y_)_1−x/4_O_3_ (PLZT) where the A-site vacancies (V_Pb_) generated by the substitution of La^3+^ for Pb^2+^ promotes the disorder of the system. Summarizing, there are a significant number of RF where different contributions to the system disorder co-exist, all of them endorsing the relaxor behavior^7,8^.

Notwithstanding the number of RF systems, their main feature arises from the existence of nanoscale polar inhomogeneities with randomly distributed directions of the dipolar moment, known as polar nanoregions (PNRs)^9^, which have been extensively confirmed by practically all characterization techniques used in material science, such as: transmission electron microscopy^10^, Raman scattering^11^, nuclear magnetic resonance^12^, neutron-scattering pair distribution functions^13^, time resolved piezoresponse force microscopy^14^, and diffuse scattering^15^. The emergence of the PNRs is related to disordered chemical inhomogeneities that unavoidably exist in these materials such as Pb^2+^ or O^2−^ vacancies, antisite ions (e.g. Nb/Mg arrangement in PMN), and so on^16^. The quenching of these disordered chemical inhomogeneities leads to the development of the disordered state being the time-response to switch their polarization vector to follow the applied external stimuli (usually applied electric fields) defined as the relaxation time (*τ*).

Both RF and SCL share two relevant common features: (i) the existence of small regions governing the relaxation processes (i.e., the cooperatively rearranging regions (CRRs) in SCL, and the PNRs in RF) as well as (ii) the dramatic increase on the relaxation times of the CRRs and PNRs on cooling, characterized by a broadened and dispersive permittivity peak in their temperature- and frequency-dependent dielectric spectra, leading to a ‘super-Arrhenius’ (SA) behavior. The SA behavior in SCL is a consequence of the increasing interaction of the closely packed liquid molecules within the CRRs leading a huge increase in relaxation time near the glass transition temperature. On the other hand, the SA in RF is interpreted as a consequence of the formation, growing and cooperative reaction between the PNRs^7^. Upon cooling, the increase of the PNRs interaction surpass the short-range forces favoring the paraelectric state. Similar to the increasing interaction of the CRRs in SCL, the increment on the PNRs interaction leads to an enormous increases in relaxation time near to the freezing temperature *T*_*f*_. Hence, the CRRs arguments of SCL resemble the PNRs concepts in RF above their freezing temperatures, thereby suggesting that the dielectric relaxation of RFs could be understood from the dynamic of SCL. Thus, an important question emerges: What we can learn from the establish concepts developed to understand the dynamic of SCL to disentangle the dielectric relaxation in RF?

Decades of studies have engendered the prevailing conviction that the ultimate parameterization for quantifying the SA behavior of glass forming systems is possible via the Vogel-Fulcher-Tammann (VFT) equation^17^:

1 $$\tau (T)={\tau }_{0}\,{\exp }(\frac{{D}_{T}{T}_{0}}{T-{T}_{0}})$$

where *D*_*T*_ denotes the fragility strength coefficient and *T*_0_ is so-called Vogel divergence temperature (*T*_0_ < *T*_*g*_ and *T* > *T*_*g*_), being *T*_*g*_ the glass transition temperature. This relationship has been assumed as the key checkpoint for developing theories/models to describe the SA behavior in SCL, linking the relaxation time with one or more thermodynamic variables. This is the case of the Adam and Gibbs (AG) model, which is probably the most popular and accepted theory for SCL. In the AG model, the configurational entropy is linked to the relaxation time by the equation^17,18^:

2 $$\tau (T)={\tau }_{0}\,{\exp }(\frac{{\rm{\Delta }}{E}_{0}}{kT{S}_{c}(T)}),$$

where *τ*_0_and Δ*E*_0_ are the relaxation time and activation energy at high temperatures, respectively. The configurational entropy *S*_*c*_(*T*) is defined as the entropy difference between the SCL state and the crystalline phase. If the configurational entropy is extrapolated to temperatures below *T*_*g*_, it becomes equal that of the crystal at a finite temperature *T*_*K*_ (i.e., the Kauzmann temperature) where the configurational entropy tends to zero (i.e., *S*_*c*_(*T*_*K*_) → 0) establishing a real limit for the dynamic in SCL. Further extrapolation in temperature (0 < *T* < *T*_*K*_) leads to a negative entropy, which is known as the Kauzmann paradox^17^. This fact strongly supports that SA behaviour in SCL could be disentangled by assuming divergent parametrization (equation predicting the divergence at finite temperature) as the case of VFT. Despite various three-parameter equations predicting the divergent relaxation time at a finite temperature have been postulated for describing the dynamics of SCL, the selection of the more appropriate model is still unclear^19–25^.

Based on the analogy between the CRRs and the PNRs, the arguments of AG have recently brought to RF^26^, written the dielectric relaxation time of RF as the following analogous equation:

3 $$\tau (T)={\tau }_{0}\exp (\frac{{\rm{\Delta }}{E}_{0}}{kT{S}_{ex}(T)}),$$

where the extrapolated excess entropy has been introduced as the difference between the paraelectric phase (analogous to the liquid) and the ferroelectric phase (analogous to the solid), ${S}_{ex}={S}_{para}-{S}_{ferro}$. On the observable time scale, the entropy of the ordered ferroelectric phase, *S*_*ferro*_, is smaller than the entropy of the disordered paraelectric phase, *S*_*para*_, but decreases more slowly with decreasing temperature. Therefore, *S*_*ex*_ tends to vanish at some finite temperature^26^.

Considering such observation, the Kauzmann statement formulated for SCL could also be valid for RF, but the appearance of a frozen polar glass phase at a glass temperature *T*_*g*_ > *T*_*K*_ in RF avoided the “entropy crisis”^26^. This was confirmed in 1990 by Viehland *et al*.^27^ showing a flawless correspondence between the freezing temperature *T*_*f*_ and the Vogel-Fulcher temperature *T*_0_ in PMN-PT. VFT became the most acceptable and used equation to describe the dielectric relaxation in RF, even more when, first, was evidenced Volgel-Fulcher freezing in RF and, second, VFT equation was derived by considering percolation and thermodynamic arguments^28,29^. This strongly indicates that for RF the SA behaviour may be elucidated by assuming the phenomenological VFT equation contrary to the case of SCL where the dominance of VFT parametrization is under debate.

Regarding the dynamic of SCL, it is important to recall that a new model-free methodology has been recently proposed^30^. The implementation of this model-free route to 55 supercooled glass forming systems have provided valuable information to unravel the dominant parameterization in SCL, showing that the fundamental justification of the VFT relation is limited to a very specific group of glass formers with definite symmetric order. Accordingly, a fundamental question arises: Is the VFT equation the more consistent parameterization to disentangle the dielectric relaxation in RF? This is an important scientific question which require a thorough investigation. The purpose of the present paper is to answer the mentioned question. To carry out it, we have first extended the model-free approach to RF deriving a more general configurational entropy equation which can recover the VFT-type as particular case. This new approach is validated and discussed by using two canonical RF (PLZT and PMN-PT) as model systems. Based on the results, a more consistent, generalized parameterization for RF is proposed.

## Results

### Model-free route (MFR) for relaxor ferroelectrics

MFR methodology enables numerical extraction of the activation energy of vitrifying systems directly from their relaxation time *τ*(*T*) data without imposing any model *a priori*^30^. This methodology is helpful in systems where their dynamic relaxation achieve a SA pattern described by:

4 $$\tau (T)={\tau }_{0}\exp (\frac{{\rm{\Delta }}{E}_{a}(T)}{{k}_{B}T}),$$

as is the case of SCL and RF systems. MFR has been recently extended for studying the low-temperature dielectric relaxations of normal ferroelectrics^31^, but this approach has never applied before to study the dynamic of RF.

In order to determine the activation energy, the apparent enthalpy energy ${\rm{\Delta }}{H^{\prime} }_{a}(T)={\rm{\Delta }}{H}_{a}/R=d\,\mathrm{ln}\,\tau (T)/d(1/T)$ should be first numerically calculated and subsequently the activation energy is numerically extracted from the following differential equation^30^:

5 $$\frac{\partial {\Delta }{E^{\prime} }_{a}(T)}{\partial (1/T)}+\frac{{\Delta }{E^{\prime} }_{a}(T)}{(1/T)}=\frac{{\Delta }{H^{\prime} }_{a}(T)}{(1/T)}.$$

Additionally to ${\Delta }{E^{\prime} }_{a}(T)$, further analysis of ${\Delta }{H^{\prime} }_{a}(T)$ allows to predicts the possible existence of dynamic crossover temperature by using the $\mathrm{ln}\,{\Delta }{H^{\prime} }_{a}(T)$ versus 1/*T* plot^30^, which give rises equivalent predictions than the Stickel *et al*.^32^ plot (supplementary information).

The non-linearity of the SA behavior is quantified by using the Grüneisen temperature index that is numerically calculated as^30,33^:

6 $${I}_{N}(T)=-\frac{d\,\mathrm{ln}\,{\rm{\Delta }}{E}_{a}(T)}{d\,\mathrm{ln}\,T}.$$

The implementation of this methodology for SCL, ranging from low molecular weight liquids, polymers liquid crystal, plastic crystals and spin glasses, elucidated three novel conclusions: (i) a simple universal linear pattern such that ${I}_{N}^{-1}(T)=aT+b$, being *a* ≠ 0 and *b* ≠ 0, (ii) a clear prevalence for the relaxation time parameterization associated with the finite-temperature divergence, *T*_*N*_ > 0, determined by the extrapolation ${I}_{N}^{-1}(T={T}_{N})=0$, showing a coincidence between the Kauzmann temperature and *T*_*N*_, (iii) the power exponent values of the derived configurational entropy equation, $n=-\,(1/b)={I}_{N}^{-1}(T=0)$, gives rise the conclusions that VFT will be valid only for a very specific group of glass formers (i.e., for *n* = 1).

Considering the SA behavior manifests in RF, and the formal similarity of the Grüneisen parameter previously noted by Samara and Boatner^34^, MFR methodology may provide valuable information related to dielectric relaxation in RF. In order to quantitatively evaluate the dynamic of the RF systems, dielectric spectroscopy measurements were performed to explore the temperature dependence of the relaxation time in (Pb_0.91_La_0.09_)(Zr_0.35_Ti_0.65_)O_3_ (hereafter labeled as PLZT). In addition, the dielectric relaxation data of 0.9PbMg_1/3_Nb_2/3_O_3_-0.1PbTiO_3_ (hereafter labeled as PMN-PT) reported by Viehland *et al*.^27^ is used here to validate the MRF procedure with a well-known data of RF behavior. Both PLZT and PMN-PT are recognized as canonical-prototype of RF.

**Figure 1:**
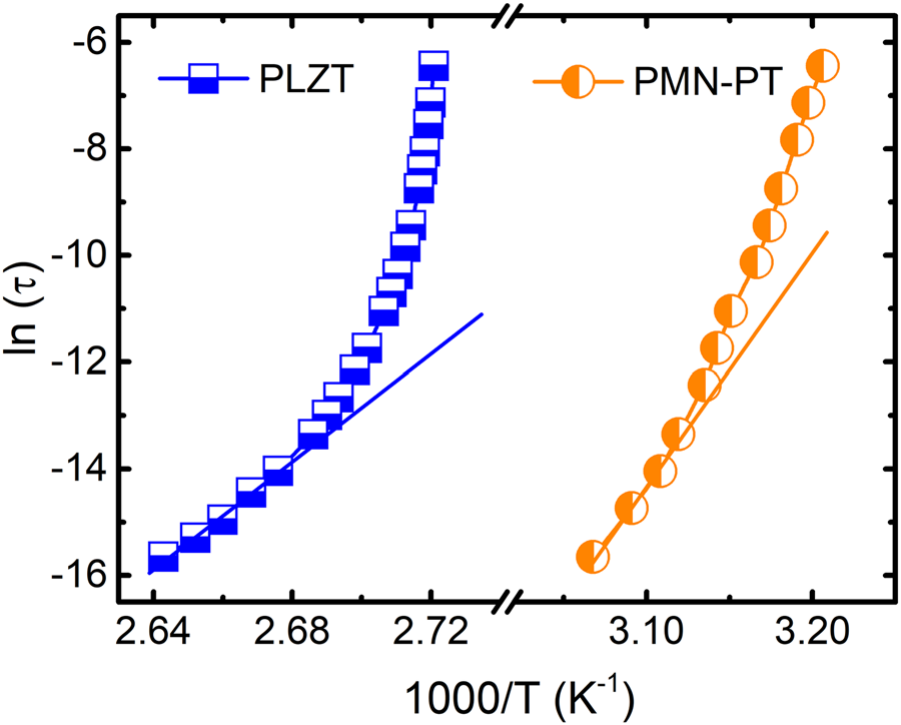
Arrhenius plot. Logarithm of the reciprocal of the measurement frequency as a function of the inverse of the temperature corresponding to the maximum value of the real permittivity. A clear non-Arrhenius (usually called super-Arrhenius) behavior is manifested as a shift from the linear Arrhenius behavior, which is exhibited by the lines drawn from high to low temperatures.

**Figure 2:**
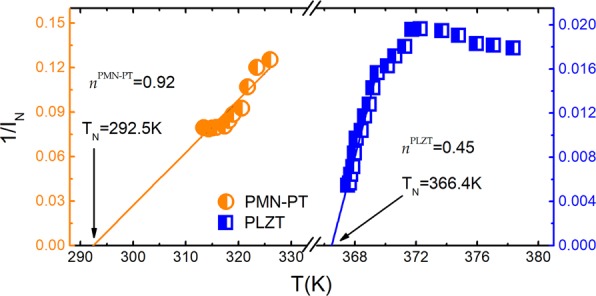
Reciprocal of the Grüneisen temperature index. Temperature evolution of the reciprocal of the index obtained directly from the experimental data for the tested materials. Only the low temperature region is considered for the linearization of the reciprocal of the index for the PLZT because two dynamical regions are evidenced (see supplementary information). The values of the divergence temperature *T*_N_ and the order parameter *n* are shown for both materials.

The temperature-dependent relaxation times of the studied RF are shown in Fig. 1. The SA behavior manifests as a shift from the Arrhenius linear behavior. Figure 2 shows the results obtained for the inverse of the index ${I}_{N}^{-1}(T)$ by developing the MFR. Four important results may be identified: (i) both RF systems exhibit a linear temperature dependence of the inverse of the index (${I}_{N}^{-1}(T)=aT+b$) similar to the behavior of the glass forming systems; (ii) since the slopes and intercepts values are *a* ≠ 0 and *b* ≠ 0, solely linear patterns with divergence temperatures occur (*T*_*N*_ > 0), thereby excluding any divergent parameterization at T_N_ = 0 K being compatible with the work by Pirc *et al*.^26^; (iii) the calculated *T*_*N*_ values match perfectly with the freezing temperatures reported for those systems; (iv) for the case of PLZT system, the obtained value of $n={I}_{N}^{-1}(T=0)$, ${n}^{PLZT}=0.45$, is noticeably different to the VFT parametrization (*n* = 1) although for PMN-PT gives rises ${n}^{PMN-PT}=0.92$, very close to the VFT one.

The value of the *n* was previously recalled as a possible order parameter correlating the dynamic of the SCL with their symmetry^30^. For instance, low values of *n* (i.e., *n*~0.2) was ascribed to materials with positional symmetry (e.g., plastic crystals) while values of *n*~1 (i.e., VFT parameterization) was attributed to no-symmetry materials (e.g., super-cooled low-molecular-weight liquids)^30^. Based on the results obtained for the SCL it is possible to wonder if there is a similar correlation for RF systems. The different values of the *n* for PLZT and PMN-PT (*n*^*PLZT*^ = 0.45 and *n*^*PMN*−10*PT*^ = 0.92) could be related to the different chemical compositions of the studied materials. Both compositions are complex perovskite-structured solid solutions presenting chemical inhomogeneities with non-isovalent ions fully or partially disordered. However, both are different from the stoichiometric point of view. The PMN-PT is a stoichiometric solid solution, where the chemical inhomogeneities are fixedly randomly distributed, forbidding the rearrangement. As a consequence of their different ionic radii, the three cations (Mg^2+^, Nb^5+^ and Ti^4+^) on the B-site of the perovskite structure generate chemical heterogeneous nanoregions for minimizing the elastic energy. These regions are the nucleus of the PNRs, which are frozen (quenched) at relatively high temperatures (below Burns temperature). Otherwise, the PLZT is a non-stoichiometric solid solution where the substitution of La^3+^ for Pb^2+^ ions leads to the creation of A-site vacancies, i.e., lead vacancies (${V}_{Pb}^{2-}$), which may rearrange below the Burns temperature.

Taking into account the analogies between RF and SCL and that different *n* values are obtained for RF systems with different compositional heterogeneity order, the following question emerge: Could be the *n* value considering as an order parameter for RF?

### Generalized VFT equation and scaling functions

In order to answer the above question, the connection between the *n* value and the configurational entropy should be explored. Considering the validity of Adam-Gibs (AG) model and the index temperature pattern, ${I}_{N}^{-1}(T)=aT+b$, a generalized configurational entropy can be derived for RF as follow (see supplementary information):

7 $${S}_{c}(T)={S}_{0}[{(1-\frac{{T}_{N}}{T})}^{n}],$$

being *S*_0_ the configurational entropy at high temperatures ($\mathop{\mathrm{lim}}\limits_{T\to \infty }{S}_{C}={S}_{0}$) and *T*_*N*_ = −*b*/*a* the divergence temperature ($\mathop{\mathrm{lim}}\limits_{T\to {T}_{N}}{S}_{C}=0$). Note that *n* = *b*^−1^ become an exponent for the configurational entropy equation. From the AG theory, the apparent activation energy can be obtained as:

8 $${\rm{\Delta }}{E}_{a}(T)={\rm{\Delta }}{E}_{0}[{(1-\frac{{T}_{N}}{T})}^{-n}],$$

where Δ*E*_0_ defines the activation energy at higher temperatures *T* ≫ *TN*.

Recalling Eq. (4), a new and more general parameterization can be obtained as:

9 $$\tau (T)={\tau }_{0}\exp [(\frac{{\rm{\Delta }}{E}_{0}}{{k}_{B}})(\frac{{T}^{n-1}}{{(T-{T}_{N})}^{n}})],$$

where the new parameter *n* emerges. For the particular case of *n* = 1, the well-known VFT equation can be recovered.

**Figure 3:**
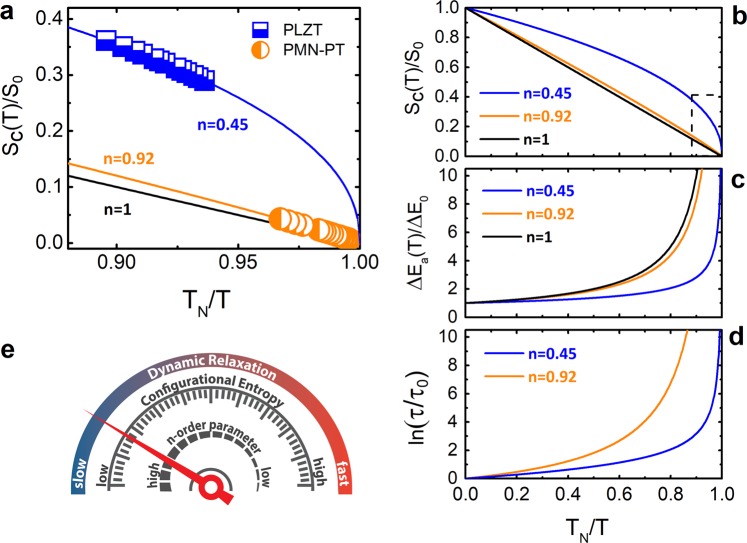
Scaling configurational entropy, activation energy and relaxation time plots. (**a**) Experimental evolution of the normalized configurational entropy as a function of the dimensionless temperature *T*_N_/*T* for the tested materials. Solid lines indicate the analytical evolution of the normalized configurational entropy computed taking into account the divergence temperature *T*_N_ and the order parameter *n* for each tested material. The analytical representation of the normalized configurational entropy for the case of *n* = 1 is also shown. (**b**–**d**) Normalized configurational entropy, activation energy and relaxation time derived from the model-free route as a function of the dimensionless temperature for the tested materials. (**e**) Schematic representation of the relation between the physical magnitudes and the dynamics of the system.

Defining a dimensionless temperature *x* = *T*_*N*_/*T*, Eqs (7–9) can be re-written as:

10 $$\frac{{S}_{c}(T)}{{S}_{0}}=[{(1-x)}^{n}],$$

11 $$\frac{{\rm{\Delta }}{E}_{a}(T)}{{\rm{\Delta }}{E}_{0}}=[{(1-x)}^{-n}],$$

12 $$\mathrm{ln}\,\tau (T)=\,\mathrm{ln}\,{\tau }_{0}+(\frac{{\rm{\Delta }}{E}_{0}}{{k}_{B}{T}_{N}})(\frac{x}{{(1-x)}^{n}}),$$

which are plotted in Fig. 3 in order to gain new insights into the physical meaning of the exponent *n*. The low temperature evolution of the configurational entropy S_c_ = *S*_*para*_ − *S*_*ferro*_ for the tested relaxors are plotted in Fig. 3a. The lines in the figures are the scaling plots functions (Eq. (10)) evaluated for the *n*-exponent values computed from the MFR methodology. The VFT case (black line) is also plotted to illustrate their inconsistency for describing the PLZT behavior.

Important conclusions can be elucidated form the scaling function in Fig. 3. The dynamic relaxation of the PLZT is faster than the PMN-PT one (Fig. 3e) indicating that the PNRs in PLZT require lower activation energy to relax (Fig. 3d) and consequently they follow the direction of the electric field easier. This leads to consider that PLZT owns a higher ferroelectric order (lower *S*_*ferro*_). This statements is clearly showed in Fig. 3c where higher (lower) values of *S*_*C*_ are ascribed to lower (higher) values of the *n*-exponent suggesting higher (lower) ferroelectric order. Undoubtedly the *n*-parameter can be considered as an indicator of the ferroelectric order in RF. It is important to remark that the avoidance of ferroelectric order is often considered a direct consequence of the frustration, arising from the competing many-body-interactions of the PNRs^35–38^.

**Figure 4:**
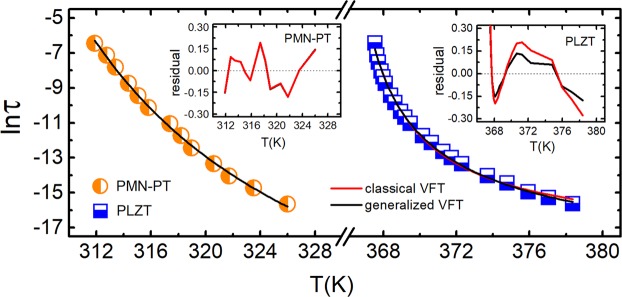
Fitting comparison between the classical and the generalized VFT equations. Dielectric relaxation data are fitted with both classical and generalized VFT equations. Residuals resulting from the fitting are plotted into inserts for both tested materials. Generalized VFT turns out to be an appropriate equation for parameterizing the dielectric relaxation of relaxor ferroelectrics.

In order to visualize our findings in *τ*(*T*) representation, a fitting comparison among the generalized VFT equation (Eq. (9)) and the classical VFT one is performed and plotted in Fig. 4. Results show that both classical and generalized VFT parameterizations fit very well with the PMN-PT relaxation data. However, the PLZT data fits better with the generalized VFT equation. This fact has a direct relation with the value of the order parameter *n*. When *n* ~ 1 (as in the case of PMN-PT) a classical VFT parameterization can be used as a particular case of Eq. (9) but a more general equation is required to obtain an accurate parameterization when the order parameter is clearly *n* ≠ 1 (as in the case of PLZT). Certainly, the proposed generalized VFT equation is a really consistent way to parameterize the dielectric relaxation in RF, regardless of the compositional heterogeneity order. More information about the fitting parameters can be found in the supplementary information.

## Discussion

Generalized VFT equations with fractional exponent have been previously proposed for describing dielectric relaxation of SCL^39,40^. However, results of the present work show that the use of a fixed fractional exponent for generalizing the VFT equation is not a proper approach for describing the dielectric relaxation in RF. The exponent *n*, which is obtained here directly from the experimental data through the Grüneisen temperature index, undertakes different values depending on the compositional heterogeneity order of the tested RF. Higher value of *n* is obtained for a stoichiometric solid solution (PMN-PT), where the chemical inhomogeneities are fixedly randomly distributed, while a non-stoichiometric solid solution (PLZT) exhibits lower value of *n*. Thus, *n* becomes an order parameter directly associated with the configurational entropy of RF. Results demonstrate that the interacting relaxing entities (PNRs) of PMN-PT require more efforts to follow the applied electric field, which manifests with a higher values of the activation energy and relaxation times (i.e., slow relaxation dynamic). Otherwise, the A-site vacancies generated by the substitution of La^3+^ for Pb^2+^ in PLZT result in a lower coupling interaction among PNRs, giving rise to a fast relaxation dynamic. It seems to be that like in SCL, where the dynamic metric (fragility) is directly related with the degree of their dipolar interactions (i.e., hydrogen bonding), the dynamic metric of RF can also be directly connected with the degree of the coupling interaction between PNRs, where the new order parameter *n* could play an important role.

Adopting a hyperbolic temperature dependence for the specific heat, a configurational entropy equation for RF was postulated by Pirc *et al*.^26,41^. Here, based on the Adam-Gibbs model and the index temperature pattern, a more general configurational entropy is proposed leading to a generalized VFT equation for successfully describing the dielectric relaxation of RF. Although the proposed *τ*(*T*) is an apparent four-parameter equation, the order parameter *n* and the divergence temperature *T*_*N*_ are obtained directly from the experimental data from a model-free route. The *n*–parameter and the temperature *T*_N_ are directly determined from the slope and the intercept of the inverse of temperature index $({I}_{N}^{-1})$ versus temperature plot through a linear fitting. It is important to point out that both parameters are intrinsically connected but are independent to the other two parameter (*τ*_0_ and Δ*E*_0_) of the generalized VFT equation. Therefore, only two parameters are extracted from the fitting of *τ*(*T*). This unbiased, model-free approach could be a powerful tool to gain knowledge about the structural origin of the RF behavior through an order parameter. Finally, the implemented methodology may open a new pathway to disentangle relaxation phenomena in other relaxor ferroics.

## Methods

### Materials

Polycrystalline lead lanthanum zirconate titanate (PLZT), with nominal composition (Pb_0.91_La_0.09_)(Zr_0.35_Ti_0.65_)O_3_, is taken as a model relaxor ferroelectric with aliovalent cation substitution. PLZT was prepared by the conventional mixed oxide method as detailed in a previous work^42^. Otherwise, lead magnesium niobate-lead titanate (PMN-PT), with nominal composition 0.9Pb(Mg_1/3_Nb_2/3_)O_3_-0.1PbTiO_3_, is taken as a canonical relaxor ferroelectric having non-isovalent ions disorder. PMN-PT was prepared by other researchers as described elsewhere^43^.

### Measurements and data acquisition

A precision LCR meter (Agilent E4980A) is used to obtain the real and imaginary parts of the permittivity of unpoled PLZT at selected frequencies from 100 Hz to 1 MHz. The sample was placed in a programmable tubular oven for measurement cooling down from 550 K to room temperature. The temperature dependence of the permittivity was measured at a cooling rate of 0.2 K/min, slow enough to avoid thermal gradients inside the sample. A quadratic fitting near the maximum of the real permittivity was carried out to obtain the temperature corresponding to the maximum value of real permittivity. For simplicity, all references to this temperature are expressed as *T* in this manuscript. Taking into account the quality of the data and to avoid bias-processing, none smooth or interpolation was performed to the raw data. Each frequency *f* corresponding to each temperature *T* serves as the metric for the relaxation time via $\tau ={(2\pi f)}^{-1}$. The PMN-PT data were taken from the Vielhand *et al*.^21^ work.

## Supplementary information

Supplementary Information

## Data Availability

The data that support the findings of this study are available from the corresponding author upon reasonable request.
