# Supplementary material for: The generalized Vogel-Fulcher-Tamman equation for describing the dynamics of relaxor ferroelectrics
Source: Sci Rep. 2019 Aug 27;9:12390. doi: 10.1038/s41598-019-48864-0 (PMC6711973; doi:10.1038/s41598-019-48864-0)
Supplement: Supplementary file 1 — Supplementary Information [file 41598_2019_48864_MOESM1_ESM.pdf]

SUPPLEMENTARY INFORMATION

The generalized Vogel-Fulcher-Tamman equation for describing the dynamics of relaxor ferroelectrics

Rafael Levit[1], Julio C. Martínez-García[2]*, Diego A. Ochoa[1], and José E. García[1]*

[1] *Department of Physics, Universitat Politècnica de Catalunya - BarcelonaTech, 08034 Barcelona, Spain*

[2] *Department of Physics, Universitat Autònoma de Barcelona, 08193 Bellaterra, Spain*

* e-mails: juliocesar.martinez@uab.cat, jose.eduardo.garcia@upc.edu

S1 Determination of dynamics regions from the Stickel plot

More than a decade ago, Stickel et al.[1-3] indicated that the validity of VFT equation has to be associated with the linearity of the "Stickel function":

$$\varphi(T) = \left(-\frac{d}{dT}\ln\tau\right)^{-1/2}, \qquad \text{(S1.1)}$$

which can be easily obtained directly from the experimental data.

The VFT equation relates the relaxation times with the temperature as follow:

$$\ln\tau = \ln\tau_0 + \frac{B}{T-T_0}, \qquad \text{(S1.2)}$$

such that:

$$\frac{d}{dT}\ln\tau = -\frac{B}{(T-T_0)^2}. \qquad \text{(S1.3)}$$

Thus, the Stickel function:

$$\varphi(T) = \frac{T}{\sqrt{B}} - \frac{T_0}{\sqrt{B}}, \qquad \text{(S1.4)}$$

turns out to be a linear temperature-dependent function.

Consequently, the Stickel plot became a key tool for estimating the so-called dynamic crossover temperature between two dynamic domains. The application of such analysis showed that at least two VFT equations are desired for describing "previtrificational" slowing down in a broader range of temperatures. Therefore, the Stickel plot represents a valuable tool

for predicting the crossover temperature from a simple linearization of the classical VFT equation.

The computed φ(T) as a function of the temperature for the studied PLZT relaxor ferroelectric is showed in Fig. S2. Two dynamic domains are identified, which are in accordance with the two dynamic regions identified in the reciprocal of the Grunieesen temperature index plot (i.e., Fig. 2 of the main manuscript).

Note that only one dynamic domain is evidenced from the Stickel plot whether the dielectric relaxation data fit very well to the VFT equation. Therefore, no crossover can be revealed for PMN-PT as shown in Fig. 2 of the main manuscript.

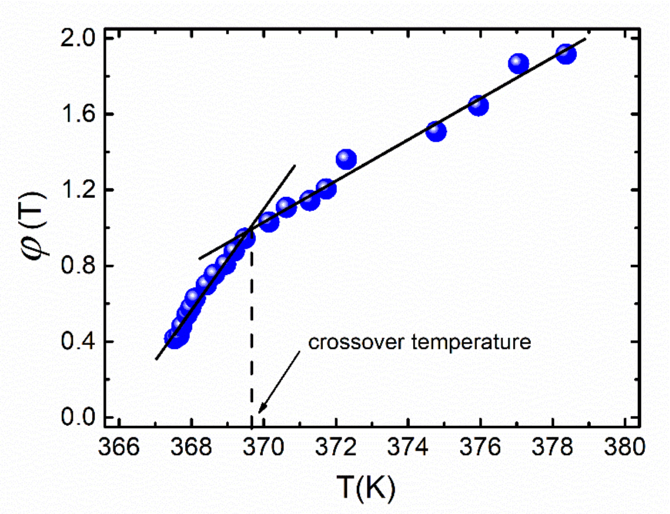

Figure S1 | Stickel plot. Stickel function versus temperature for the tested PLZT material. Two linear regions for φ(T) may be easily identified. A linear fitting for the two linear patterns are plotted. The crossover temperature between the two dynamic regions can be estimated as indicated.

S2 Generalized entropy equation for relaxor ferroelectrics

The Adam-Gibbs theory relates the apparent activation energy and the configurational entropy such that:[4]

$$\Delta E_a(T) \propto \frac{1}{S_c(T)}. \quad \text{(S2.1)}$$

Considering the validity of Adam-Gibbs model, we can write:

$$\ln \Delta E_a(T) = ct - \ln S_c(T). \quad \text{(S2.2)}$$

Finding a derivative of equation (S2.1) allows to obtain:

$$\frac{\partial \ln \Delta E_a(T)}{\partial T} = -\frac{1}{S_c(T)}\frac{\partial S_c(T)}{\partial T}. \tag{S2.3}$$

Considering the definition of the temperature index:[5]

$$I_N(T) = -\frac{\partial \ln \Delta E_a(T)}{\partial \ln T} = -T\frac{\partial \ln \Delta E_a(T)}{\partial T}, \tag{S2.4}$$

and substituting equation (S2.3) in equation (S2.4), we obtain:

$$\frac{I_N(T)}{T} = \frac{1}{S_c(T)}\frac{\partial S_c(T)}{\partial T}. \tag{S2.5}$$

Integrating equation (S2.5) from $T_0 \to \infty$ to a finite temperature T,

$$\int_{\infty}^{T} \frac{I_N(T)}{T} dT = \ln\left[\frac{S_c(T)}{S_c(\infty)}\right], \tag{S2.6}$$

and adopting a linear behavior for the inverse of the index,

$$\frac{1}{I_N(T)} = aT + b, \tag{S2.7}$$

we can obtain, substituting equation (S2.7) in equation (S2.6), the following equation:

$$\int_{\infty}^{T} \frac{1}{T(aT+b)} dT = \ln\left[\frac{S_c(T)}{S_c(\infty)}\right]. \tag{S2.8}$$

Calculating the primitive in equation (S2.8):

$$\ln\left(T^{\frac{1}{b}}(aT+b)^{-\frac{1}{b}}\Big|_{\infty}^{T}\right) = \ln\left[\frac{S_c(T)}{S_c(\infty)}\right], \tag{S2.9}$$

and after some math transformations, we can write:

$$\ln\left(\left(a+\frac{b}{T}\right)^{-\frac{1}{b}}\Bigg|_{\infty}^{T}\right) = \ln\left[\frac{S_c(T)}{S_c(\infty)}\right]. \tag{S2.10}$$

After evaluating the integral in equation (S2.10):

$$\ln\left(a+\frac{b}{T}\right)^{-\frac{1}{b}} - \ln(a)^{-\frac{1}{b}} = \ln\left[\frac{S_c(T)}{S_c(\infty)}\right], \tag{S2.11}$$

we can obtain:

$$\ln\left[\frac{(a)^{-\frac{1}{b}}\left(1+\frac{b}{aT}\right)^{-\frac{1}{b}}}{(a)^{-\frac{1}{b}}}\right] = \ln\left[\frac{S_c(T)}{S_c(\infty)}\right], \tag{S2.12}$$

leading to:

$$S_c(T) = S_c(\infty)\left(1+\frac{b}{aT}\right)^{-\frac{1}{b}}. \tag{S2.13}$$

The equation (S2.13) is labeled as the generalized configurational entropy relation, which after some variable adjustments can be written in the following way:

$$S_c(T) = S_0\left(1-\frac{T_N}{T}\right)^{n}, \tag{S2.14}$$

being the configurational entropy at high temperatures defined as $S_c(\infty) = S_0$, the divergent temperature as $T_N = -b/a$ (also namely the Kauzmann temperature), and the exponent $n = -(1/b)$ as an order parameter.

Adopting a hyperbolic temperature dependence for the specific heat, a configurational entropy equation for relaxor ferroelectrics was obtained by Pirc et al.[6,7] as follow:

$$S_c(T) = S_0\left[1-\frac{T_{VFT}}{T}\right], \tag{S2.15}$$

where T_{VFT} is the Vogel-Fulcher-Tamman temperature and S_0 the configurational entropy at higher temperatures.

The configurational entropy given by the equation (S2.14) generalizes the equation (S2.15), which can be recovered for the particular case of $n = 1$ taking into account that T_{VFT} is conceptually equivalent to the divergent temperature T_N.

Assuming the validity of AG theory for relaxor ferroelectrics, the activation energy can be written from equation (S2.14) in the following way:

$$\Delta E_a(T) = \Delta E_0\left(1-\frac{T_N}{T}\right)^{-n}, \tag{S2.16}$$

where ΔE_0 defines the activation energy at higher temperatures.

Substituting equation (S2.16) in the SA relationship:

$$\tau(T)_{SA} = \tau_0 \exp\left(\frac{\Delta E_a(T)}{k_B T}\right), \tag{S2.17}$$

a more general equation for the relaxation time can be written as:

$$\ln \tau(T)_G = \ln \tau_0 + \left(\frac{\Delta E_0}{k_B T}\right)\left(1 - \frac{T_N}{T}\right)^{-n}, \quad \text{(S2.18)}$$

which after some transformations leads to:

$$\ln \tau(T)_G = \ln \tau_0 + \left(\frac{\Delta E_0}{k_B}\right)\left(\frac{T^{n-1}}{(T-T_N)^n}\right). \quad \text{(S2.19)}$$

Finally, a generalized relaxation time equation is obtained as follow:

$$\tau(T)_G = \tau_0 \exp\left\{\left(\frac{\Delta E_0}{k_B}\right)\left(\frac{T^{n-1}}{(T-T_N)^n}\right)\right\}. \quad \text{(S2.20)}$$

For the particular case of $n = 1$, the classical VFT equation is recovering from equation (S2.20), writing the divergent temperature T_N as T_{VFT}:

$$\tau(T)_{VFT} = \tau_0 \exp\left(\frac{\Delta E_0}{k_B(T - T_{VFT})}\right). \quad \text{(S2.21)}$$

S3 Fitting parameters of the relaxation data

Table S1 shows the values of the fitting parameters obtained from the fitting of the relaxation data of the studied materials by using both classical and generalized VFT functions given by equations (S2.21) and (S2.20), respectively. The fitting curves are shown in Fig. 4 of the main manuscript.

Table S2 | Parameters of the relaxation data. Values of the fitting parameters $\ln \tau_0$ and $\Delta E_0/k_B$ obtained from the fitting of the relaxation data. The values of the parameters n and T_N are obtained previously from the model-free route (see Fig. 2 of the main manuscript). The resulting chi-square (χ^2) are tabulated as a metric of the fitting quality.

	Equation	$\ln \tau_0$	$\Delta E_0/k_B$	n	T_N[K]	$\chi^2 * 100$
**PLZT**	Classical VFT	-14.09(0.18)	33.21(1.57)	1	365(0.04)	3.21
	Generalized VFT	-17.44(0.11)	459.69(5.9)	0.45	366(0.01)	1.77
**PMN-PT**	Classical VFT	-25.58(1.44)	458.16(71)	1	292(1.78)	1.64
	Generalized VFT	-26.24(1.59)	560.16(84)	0.92	293(1.71)	1.65

Supplementary References

1. Stickel, F., Fischer E. W. & Richert R. Dynamics of glass-forming liquids. I. Temperature-derivative analysis of dielectric relaxation data. *J. Chem. Phys.* **102**, 6251-6257 (1995).
2. Stickel, F., Fischer, E. W. & Richert, R. Dynamics of glass-forming liquids. II. Detailed comparison of dielectric relaxation, dc-conductivity, and viscosity data *J. Chem. Phys.* **104**, 2043-2055 (1996).
3. Hansen, C., Stickel, F., Berger, P., Richert, R. & Fischer, E. W. Dynamics of glass-forming liquids. III. Comparing the dielectric α- and β-relaxation of 1-propanol and o-terphenyl. *J. Chem. Phys.* **107**, 1086-1093 (1997).
4. Adam, G. & Gibbs, J. H. On the temperature dependence of cooperative relaxation properties in glass-forming liquids. *J. Chem. Phys.* **43**, 139–146 (1965).
5. Dyre, J. C. & Olsen, N. B. Landscape equivalent of the shoving model. *Phys. Rev. E* **69**, 042501 (2004).
6. Pirc, R. & Blinc, R. Freezing dynamics of relaxor ferroelectrics and dipolar glasses. *Ferroelectrics* **379**, 30–34 (2009).
7. Pirc, R. & Kutnjak, Z. Electric-field dependent freezing in relaxor ferroelectrics. *Phys. Rev. B* **89**, 184110 (2014).
